# Atopic dermatitis and hand eczema in Danish adults: A nationwide population‐based study

**DOI:** 10.1111/cod.14691

**Published:** 2024-09-12

**Authors:** Anna Schultz Vinge, Lone Skov, Jeanne Duus Johansen, Anna Sophie Quaade

**Affiliations:** ^1^ Department of Dermatology and Allergy Copenhagen University Hospital – Herlev and Gentofte Copenhagen Denmark; ^2^ Department of Clinical Medicine, Faculty of Health and Medical Sciences University of Copenhagen Copenhagen Denmark; ^3^ The National Allergy Research Centre, Department of Dermatology and Allergy Copenhagen University Hospital – Herlev and Gentofte Copenhagen Denmark

**Keywords:** atopic dermatitis, contact allergy, epidemiology, hand eczema, prevalence

## Abstract

**Background:**

Atopic dermatitis (AD) and hand eczema often co‐occur, particularly among adults.

**Objectives:**

To examine the interplay between AD and hand eczema in the general population, by characterising prevalence, disease severity, contact sensitization, and comorbidities in individuals with one or both conditions.

**Materials and Methods:**

In this cross‐sectional study, 100 000 randomly selected adults in the Danish general population received a questionnaire via a secure, digital mailbox linked to their civil registration number. Participants answered questions regarding eczema, disease severity, patch testing, and comorbidities.

**Results:**

A total of 40 007 individuals responded to the question on a lifetime prevalence of AD, and the prevalence among adult Danes was 9.0%. Nearly one third of individuals with AD reported moderate to severe disease. AD was associated with contact sensitization and increased hand eczema prevalence. Individuals with both AD and hand eczema reported worse disease severity. Furthermore, having both conditions was associated with an increased risk of psychiatric comorbidities.

**Conclusions:**

This study provided updated information about unselected adults with AD in Denmark. Individuals with both AD and hand eczema represent a vulnerable subgroup that physicians should be attentive to.

## Graphical abstract

In this cross‐sectional questionnaire‐based study, the interplay between AD and hand eczema was examined in the adult, Danish general population. Individuals with both AD and hand eczema represent a vulnerable subgroup that physicians should be attentive to. Graphical abstract created with BioRender.com. AD, atopic dermatitis.

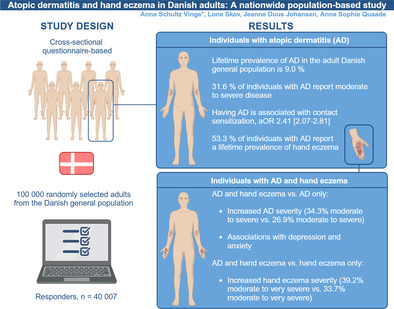

## 1 INTRODUCTION

Atopic dermatitis (AD) is a common inflammatory skin disease affecting up to 10% of adults in high‐income countries.^1^ The anatomical distribution varies with different age groups; adults typically have lesions affecting the face, neck, flexures, or hands.^1^ Severity of AD in adult general population settings is sparsely described in the literature. In a questionnaire‐based multinational study, 10%–21% of the responders with AD reported severe disease.^2^ Having AD is associated with a high disease burden^3^ and psychiatric comorbidities such as depression or anxiety.^4^ A recent study by Silverberg et al. showed that individuals with AD experience worse disease severity and health‐related quality of life if the face, neck, or hands are involved.^5^ Whether AD is associated with an increased risk of contact sensitization is still an ongoing topic of debate.^6^

Hand eczema is an umbrella term covering different clinical subtypes, for example, hyperkeratotic palmar hand eczema and pulpitis, and different aetiological subtypes, for example, irritant contact dermatitis, allergic contact dermatitis, and atopic hand eczema.^7^, ^8^ Since hand eczema can be a part of the clinical presentation of AD, and AD increases the risk of developing hand eczema,^9^, ^10^ AD and hand eczema often co‐occur—particularly among adults. In a meta‐analysis on hand eczema prevalence in the general population, one third of individuals with hand eczema reported a history of AD.^11^ Another meta‐analysis found that unselected individuals with AD had an increased odds ratio (OR) of 5.7 of having hand eczema compared with those without AD.^10^

In a recent large cross‐sectional study investigating chronic hand eczema and its impact on quality of life among adults in the Danish general population, we found that impairment of health‐related quality of life was associated with severity of hand eczema.^12^ Of note, when examining individuals with current hand eczema, having a lifetime prevalence of AD was associated with a moderate to very strong reduction in health‐related quality of life, even after adjusting for confounders. This gave the incentive to study the interplay between AD and hand eczema more in detail using the above population data. The aim was to characterise persons with one or both conditions in relation to prevalence, disease severity, comorbidities, and contact sensitization to identify subgroups that would potentially benefit from increased medical attention.

## 2 MATERIALS AND METHODS

This cross‐sectional study was based on a questionnaire that was sent to 100 000 randomly selected adults (18–75 years of age) in the Danish general population from May to June 2021. They were contacted via a secure, digital mailbox linked to their civil registration number (e‐Boks). For additional information regarding the questionnaire, please refer to the recent publication by Quaade et al.^12^ The study was approved by the Danish Data Protection Agency (P‐2020‐558) and the Danish Health Data Authority (FSEID‐00005217).

Self‐reported lifetime prevalence of physician‐diagnosed AD was determined based on the question ‘has a doctor ever told you, or your parents, that you have/had atopic eczema (childhood eczema, asthmatic eczema)?’. This question is a modified version of the validated question from Silverberg et al. with a sensitivity of 0.43 and a specificity of 0.97.^13^ No AD was defined as answering either ‘no’ or ‘unknown’. An affirmative answer led to the question ‘do you still have AD?’. Point prevalence was defined as ‘yes, currently’, and 1‐year prevalence was defined as either ‘yes, currently’ or ‘not currently, but within the past year’. Individuals with a 1‐year prevalence of AD were asked to evaluate current severity of the disease with the validated tool Patient‐Oriented SCORing for AD (PO‐SCORAD).^14^ The tool elicits self‐assessment of lesion severity and evaluation of subjective symptoms (sleep disturbance and pruritus), resulting in a score between 0 and 103. Severity strata for PO‐SCORAD was previously tested in a population‐based cohort, suggesting mild AD was determined by a score ≤27, moderate disease between 28 and 56, and severe AD as a score >56.^15^

Questions regarding patch testing and hand eczema prevalence have been described by Quaade et al.^12^ For self‐assessment of hand eczema disease severity, responders were presented with both the validated photographic guide^16^ and a visual analogue scale.

The questionnaire included questions regarding cardiovascular, psychiatric, and atopic comorbidities, socioeconomic status, smoking, alcohol intake, sick leave, general practitioner consultations, and overall health ratings. Demographic details including information on sex and age, were retrieved from the civil registration system.^17^

Localization of eczema in other body areas than the hands was determined by the question ‘where on the body do you have/have you had eczema?’. For each specific body area (face and ears, scalp, neck and upper chest, armpit, arms, hands, abdomen, inguinal area, genitalia, back, buttocks, legs, and feet) responders could reply with either ‘never’, ‘currently’, ‘within the last 12 months’, or ‘for more than 12 months ago’.

### 2.1 Statistical analysis

All statistical analyses were performed with IBM SPSS Statistics, version 29.0.1.0 (IBM Corp., Armonk, NY, USA). Missing answers were excluded from statistical analysis (see Appendix S1). All numeric variables did not show a Gaussian distribution. Both medians (interquartile range [IQR]) and means ± standard deviation [SD] are presented due to the large sample size. Differences between group medians were calculated using Mann–Whitney *t* test and between group means using student's *t*‐test. Categorical variables were presented as numbers and proportions (%). Differences between group proportions were calculated using Chi‐square test. If an overall significant difference was found, post hoc testing with pairwise *Z*‐tests based on adjusted standardised residuals was performed. To investigate the association between educational attainment and hand eczema, the association between contact sensitization and AD, and the association between cardiovascular or psychiatric comorbidities and hand eczema, binary logistic regression analyses were performed. Results were presented as crude and adjusted (a) OR with 95% confidence intervals (CI). Bar plots were created using GraphPad Prism version 10.1.2 (La Jolla, CA, USA) and figures were created with BioRender.com.

## 3 RESULTS

Overall response rate on the questionnaire was 42.7%. Characterisation of responders and non‐responders have previously been described.^12^ A total of 40 007 individuals responded to the question on a self‐reported lifetime prevalence of physician‐diagnosed AD. These responders constitute the study population and are presented in Table 1 (for an extended version of the table including information on municipality group, personal income, educational level, smoking status, alcohol intake, severity of hand eczema and AD, sick leave, and health care utilisation, see Table S1).

**TABLE 1:** Characteristics of responders stratified by self‐reported lifetime prevalence of physician‐diagnosed atopic dermatitis.

	Total (*n* = 40 007)	Atopic dermatitis (*n* = 3601)	No atopic dermatitis (*n* = 36 406)	*p*‐Value
Age, median (IQR)	54.7 (25)	42.4 (24)	55.8 (24)	**<0.001**
Sex, females, *n* (%)	22 680 (56.7)	2705 (75.1)	19 975 (54.9)	**<0.001**
Comorbidities^a^, *n* (%)				
Asthma	5702 (14.3)	1173 (32.6)	4529 (12.5)	**<0.001**
Allergic rhinitis	8659 (21.7)	1509 (42.0)	7150 (19.7)	**<0.001**
Diabetes	2130 (5.3)	80 (2.2)	2050 (5.6)	**<0.001**
Hypertension	10 117 (25.3)	574 (15.9)	9543 (26.2)	**<0.001**
Hypercholesterolemia	8749 (21.9)	477 (13.2)	8272 (22.7)	**<0.001**
Myocardial infarction	719 (1.8)	19 (0.5)	700 (1.9)	**<0.001**
Stroke	987 (2.5)	45 (1.2)	942 (2.6)	**<0.001**
Depression	4978 (12.4)	665 (18.5)	4313 (11.8)	**<0.001**
Anxiety	3351 (8.4)	521 (14.5)	2830 (7.8)	**<0.001**
Stress	6434 (16.1)	824 (22.9)	5610 (15.4)	**<0.001**
Hand eczema prevalence, *n* (%)				
Lifetime	9081 (22.9)	1915 (53.3)	7166 (19.8)	**<0.001**
1 year	4928 (12.4)	1206 (33.7)	3722 (10.3)	**<0.001**
Point	2159 (5.5)	610 (17.1)	1549 (4.3)	**<0.001**
Ever patch tested, *n* (%)	7375 (18.7)	1398 (39.5)	5977 (16.7)	**<0.001**
Positive patch test	4101 (60.6)	989 (76.0)	3112 (57.0)	**<0.001**
Of patch‐tested individuals, *n* (%)				
≥2 positive allergens from patch test	1033 (15.3)	343 (26.4)	690 (12.6)	**<0.001**
Of patch tested individuals, *n* (%)				
Positive allergen				
Of patch‐tested individuals, *n* (%)				
Nickel	1337 (19.8)	360 (27.7)	977 (17.9)	**<0.001**
Chrome	231 (3.4)	74 (5.7)	157 (2.9)	**<0.001**
Cobalt	177 (2.6)	58 (4.5)	119 (2.2)	**<0.001**
Perfume	816 (12.1)	263 (20.2)	553 (10.1)	**<0.001**
Colourants	284 (4.2)	120 (9.2)	164 (3.0)	**<0.001**
Preservatives	384 (5.7)	120 (9.2)	264 (4.8)	**<0.001**
Acrylates	31 (0.5)	8 (0.6)	23 (0.4)	0.353
Epoxy	64 (0.9)	15 (1.2)	49 (0.9)	0.392
Rubber chemicals	236 (3.5)	82 (6.3)	154 (2.8)	**<0.001**
Colophonium	72 (1.1)	19 (1.5)	53 (1.0)	0.122
Others/do not remember which allergy	2038 (30.1)	573 (44.0)	1465 (26.8)	**<0.001**

*Note*: No atopic dermatitis was defined as the individuals responding ‘No’ or ‘Unknown’ to the question on a lifetime prevalence of physician diagnosed AD. For numeric variables, differences between group medians were calculated using Mann–Whitney *U* test and between group means using students *t*‐test. For categorical variables, differences between group proportions were calculated using Chi‐square test. If an overall significant difference was found, post hoc testing with pairwise Z‐tests based on adjusted standardised residuals was performed. Missing answers were excluded and can be found in Appendix S1. Bold values denote significance at the *p* < 0.05 level.

Abbreviations: AD, Atopic dermatitis; IQR, interquartile range.

^a^ Self‐reported lifetime prevalence of physician diagnosed cardiovascular and psychiatric comorbidities.

### 3.1 Prevalence and severity of AD

The self‐reported prevalence of physician diagnosed AD in the adult, Danish general population was 9.0% (95% CI [8.7%–9.3%]) for lifetime prevalence, 3.6% (95% CI [3.4%–3.8%]) for 1‐year prevalence, and 2.0% (95% CI [1.9%–2.1%]) for point prevalence. For individuals responding ‘Yes’ to having a lifetime prevalence of AD, the 1‐year prevalence of the disease was 40.0% (95% CI [38.4%–41.6%]), and the point prevalence was 22.1% (95% CI [20.8%–23.5%]). Individuals with AD were younger and more likely to be female than those without AD (Table 1). Overall, the self‐reported severity of AD as assessed by the PO‐SCORAD was moderate to severe in 31.6% of the cases (Table S1).

### 3.2 Patch testing and contact sensitization by AD status

As presented in Table 1, the proportion of patch‐tested individuals was significantly higher in the AD group (AD 39.5% vs. no AD 16.7%, *p* < 0.001). In addition, when looking at individuals who had been patch tested, the patch test was more frequently positive and with more than one positive allergen in individuals with AD as compared with those without, Table 1. Allergens that were more frequently positive on a patch test in the AD group included nickel, chrome, cobalt, perfume, colourants, preservatives, and rubber chemicals (all *p*‐values <0.001, Table 1). Table 2 presents crude and adjusted ORs for the association between contact sensitization and AD when assessing patch‐tested individuals. When examining individuals with a lifetime prevalence of AD stratified by sex, a significantly higher proportion of females than males had previously been patch tested (females 41.2% vs. males 34.5%, *p* < 0.001). Nevertheless, no significant difference in positive patch test results among patch‐tested individuals was found (females 75.5% vs. males 77.7%, *p* = 0.444).

**TABLE 2:** Association between contact sensitization and a lifetime prevalence of atopic dermatitis.

	Included in analysis (*n*)	Crude OR [95% CI]	*p*‐Value	Included in analysis (*n*)	Model 1^a^ OR [95% CI]	*p*‐Value	Included in analysis (*n*)	Model 2^b^ OR [95% CI]	*p*‐Value
Positive patch test (of patch‐tested individuals)^c^									
Atopic dermatitis	1301	2.39 [2.08–2.75]	**<0.001**	1301	2.43 [2.10–2.80]	**<0.001**	1144	2.41 [2.07–2.81]	**<0.001**
No atopic dermatitis	5461	1		5461	1		4357	1	
≥2 positive allergens from patch test (of patch‐tested individuals)^c^									
Atopic dermatitis	1301	2.48 [2.14–2.87]	**<0.001**	1301	2.57 [2.20–3.01]	**<0.001**	1144	2.65 [2.24–3.14]	**<0.001**
No atopic dermatitis	5461	1		5461	1		4357	1	

*Note*: Binary logistic regression analyses displayed as ORs with 95% CIs for the outcome positive patch test compared with negative patch test, and more than one positive allergen from the patch test compared with having one positive allergen or a negative patch test. No atopic dermatitis was defined as the individuals responding ‘No’ or ‘Unknown’ to the question on lifetime prevalence of physician diagnosed AD. Missing answers were excluded. Information regarding missing data can be found in Appendix S1. Bold values denote significance at the *p* < 0.05 level.

Abbreviations: AD, CI, confidence interval; OR, odds ratio.

^a^ Adjusted for age and sex.

^b^ Adjusted for age, sex, educational level, and smoking. Educational level was defined as lower education (unskilled or skilled profession) or higher education (higher education <3 years, 3–4 years, or >4 years). Smoking status was divided into daily smoking or not.

^c^ 7375 individuals were previously patch tested. Of these, 1398 had a self‐reported lifetime prevalence of physician‐diagnosed atopic dermatitis, and 5977 did not. Individuals responding “Unknown” to result of patch test were excluded as missing answers (atopic dermatitis *n* = 97, no atopic dermatitis *n* = 516).

### 3.3 Comorbidities by AD status

Comorbidities according to AD status are presented in Table 1. As expected, individuals with AD reported higher prevalence of asthma and allergic rhinitis. Cardiovascular comorbidities (diabetes, hypertension, hypercholesterolemia, myocardial infarction, and stroke) were less frequent in the AD group compared with individuals without AD. This was further analysed employing binary logistic regression. After adjusting for sex, age, educational level, alcohol intake, and smoking there was no significant difference in odds for cardiovascular comorbidities between individuals with and without AD, except from diabetes (aOR 0.68, 95% CI [0.53–0.87]; Table S2).

With regards to psychiatric comorbidities, significant associations between AD and depression, anxiety, and stress were found that remained significant after adjusting for age, sex, educational level, alcohol, and smoking. For depression, aOR was 1.33, 95% CI [1.20–1.46], anxiety aOR 1.36, 95% CI [1.21–1.52], and stress aOR 1.34, 95% CI [1.22–1.47]. For crude and adjusted ORs (see Table S2).

### 3.4 Prevalence of hand eczema by AD status

Overall, 22.9% of the responders reported a lifetime prevalence of hand eczema (Table 1). This prevalence was significantly higher in individuals with AD (AD 53.3% vs. no AD 19.8%, *p* < 0.001). Similarly, the 1‐year and point prevalence of hand eczema were higher in the AD group, Table 1. Females with a lifetime prevalence of AD (*n* = 2705) reported a higher lifetime prevalence of hand eczema compared with males with AD (*n* = 896), 54.8% versus 48.8%, *p* = 0.002. The same applied to 1‐year prevalence (females 35.7% vs. males 27.6%, *p* = 0.001) and point prevalence of hand eczema (females 17.5% vs. males 15.8%, *p* = 0.029).

### 3.5 Association between educational level and hand eczema in individuals with AD

Information on educational attainment in individuals with and without AD can be found in Table S1. We examined whether there was an association between educational level and the lifetime prevalence of hand eczema in individuals with a history of AD using a logistic regression analysis, see Table 3. Lower educational level (unskilled or skilled profession) was significantly associated with a lifetime prevalence of hand eczema compared with higher educational level (higher education of <3, 3–4, or >4 years). This was still the case after adjustment for age, sex, and smoking.

**TABLE 3:** Association between educational attainment and a lifetime prevalence of hand eczema in individuals with atopic dermatitis.

	Included in analysis (*n*)	Crude OR [95% CI]	*p*‐Value	Included in analysis (*n*)	Model 1^a^ OR [95% CI]	*p*‐Value	Included in analysis (*n*)	Model 2^b^ OR [95% CI]	*p*‐Value
Lifetime prevalence of hand eczema									
Lower educational level	1890	1.23 [1.07–1.42]	**0.004**	1890	1.19 [1.03–1.38]	**0.019**	1888	1.19 [1.03–1.38]	**0.020**
Higher educational level	1238	1		1238	1		1236	1	

*Note*: Binary logistic regression analyses displayed as ORs with 95% CIs for the outcome self‐reported lifetime prevalence of hand eczema compared with no hand eczema. Educational level was defined as lower education (unskilled or skilled profession) or higher education (higher education <3, 3–4, or >4 years). Only individuals reporting a lifetime prevalence of atopic dermatitis and responding to both the question on a lifetime prevalence of hand eczema and educational level were included in analysis (*n* = 3133). Of these, *n* = 1894 reported a lower educational level, and *n* = 1239 a higher educational level. Missing answers were excluded. Information regarding missing data can be found in Appendix S1. Bold values denote significance at the *p* < 0.05 level.

Abbreviations: CI, confidence interval; OR, odds ratio.

^a^ Adjusted for age and sex.

^b^ Adjusted for age, sex, and smoking. Smoking status was divided into daily smoking or not.

### 3.6 Greater self‐reported disease severity and distribution of current eczema in individuals with both AD and hand eczema

**FIGURE 1:**
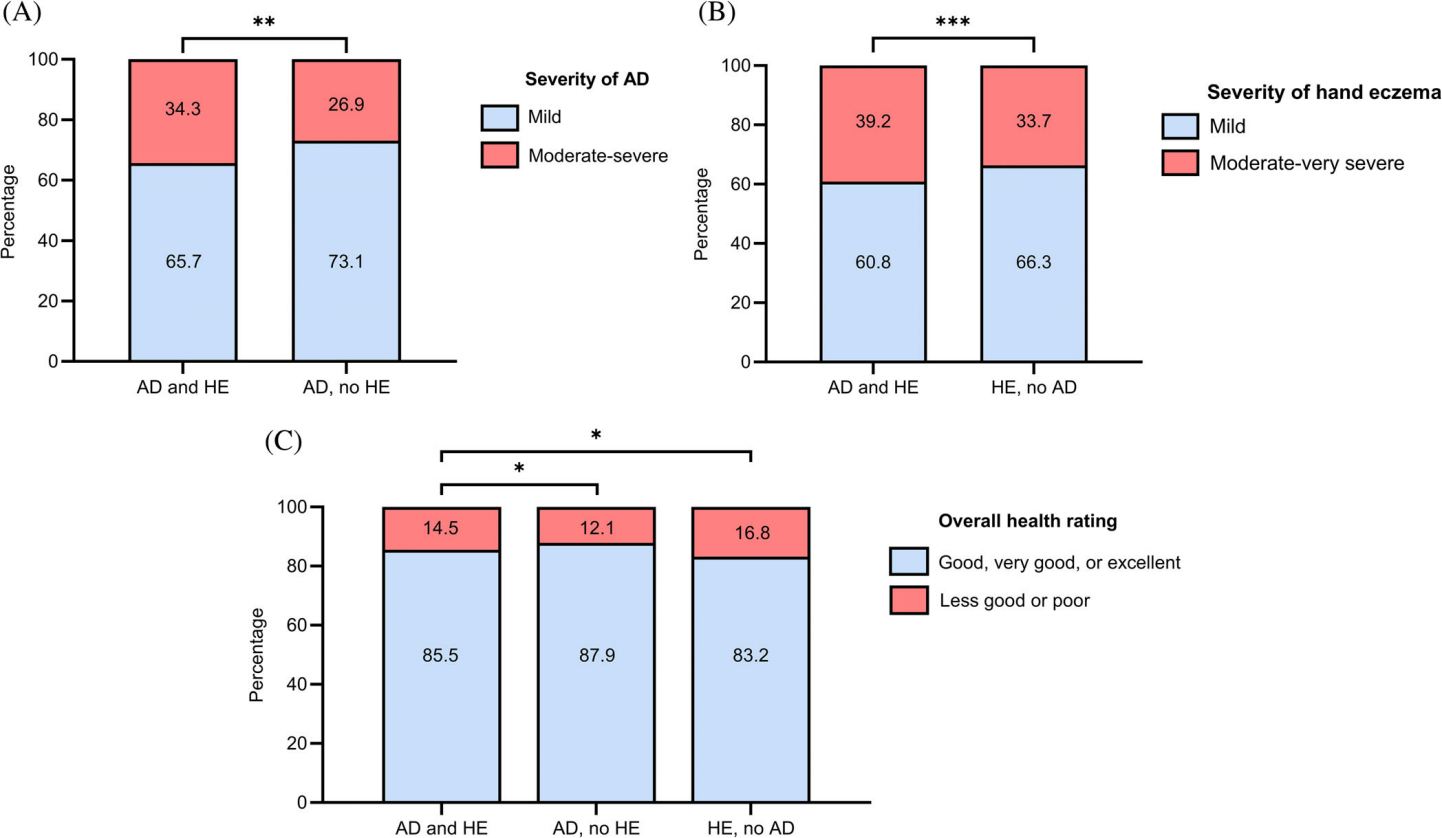
(A) Severity of current atopic dermatitis assessed by PO‐SCORAD for individuals with self‐reported 1‐year prevalence of atopic dermatitis and a lifetime prevalence of hand eczema (*n* = 883) or without a lifetime prevalence of hand eczema (*n* = 505), %. Score ≤ 27: Mild disease. Score >27: Moderate to severe disease. (B) Severity of hand eczema averagely the past year assessed by the photographic guide for individuals with a 1‐year prevalence of hand eczema with a lifetime prevalence of atopic dermatitis (*n* = 1197) or without a lifetime prevalence of atopic dermatitis (*n* = 3681), %. (C) Self‐reported overall health rating for individuals with atopic dermatitis and hand eczema (*n* = 1879), individuals with atopic dermatitis and no hand eczema (*n* = 1641), and individuals with hand eczema and no atopic dermatitis (*n* = 7036), %. The groups are divided according to self‐reported lifetime prevalence of physician‐diagnosed atopic dermatitis and self‐reported lifetime prevalence of hand eczema. Missing answers were excluded. Information regarding missing data can be found in Appendix S1. AD, atopic dermatitis; HE, hand eczema; PO‐SCORAD, Patient‐Oriented SCORing for AD. *p ≤ 0.05; **p ≤ 0.01; ***p ≤ 0.001.

**FIGURE 2:**
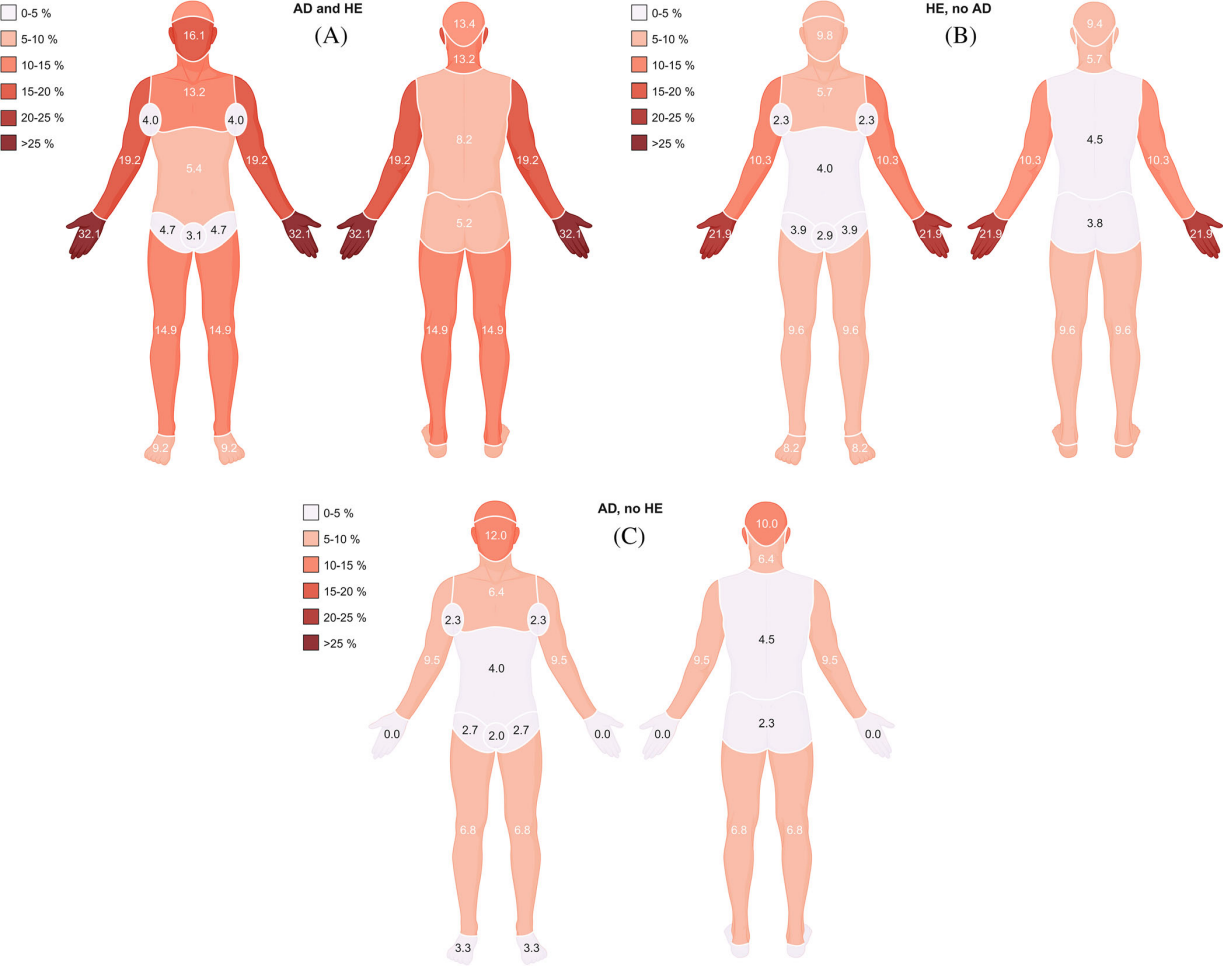
(A) Prevalence of current eczema in different anatomical sites for individuals with a self‐reported lifetime prevalence of physician‐diagnosed atopic dermatitis and a self‐reported lifetime prevalence of hand eczema (*n* = 1914), %. (B) Prevalence of current eczema in different anatomical sites for individuals with a self‐reported lifetime prevalence of hand eczema without atopic history (*n* = 7158), %. (C) Prevalence of current eczema in different anatomical sites for individuals with a self‐reported lifetime prevalence of atopic dermatitis without a lifetime prevalence of hand eczema (*n* = 1671), %. Figures created with BioRender.com. Data for the figures can be found in Table S4a,b. Missing answers were excluded. Information regarding missing data can be found in Appendix S1. AD, atopic dermatitis; HE, hand eczema.

We next stratified the population according to a lifetime prevalence of AD and hand eczema. Thus, three groups were formed: AD and hand eczema (*n* = 1915), AD without hand eczema (*n* = 1675), and hand eczema without AD (*n* = 7166). Detailed characteristics of these groups are depicted in Table S3. When investigating self‐reported disease severity of AD, a greater proportion of individuals with both AD and hand eczema reported moderate to severe AD (34.3%) compared with individuals with only AD (26.9%, *p* = 0.004, Figure 1A). When evaluating self‐reported hand eczema severity by the photographic guide, a greater proportion of individuals with both AD and hand eczema reported moderate to very severe hand eczema (39.2%) compared with individuals with only hand eczema (33.7%, *p* < 0.001, Figure 1B). Figure 2 depicts the distribution of current eczema in different anatomical sites for the three groups. Individuals with both AD and hand eczema reported current eczema to a significantly greater extent in every body area compared with individuals with only hand eczema (all *p*‐values <0.05, see Table S4a), except for the inguinal area, the genitalia, and feet. Similarly, when comparing individuals with AD and hand eczema to individuals with only AD, a significantly greater proportion had current eczema in every body area (all *p*‐values <0.05, see Table S4b), except the abdomen and genitalia.

### 3.7 Overall health rating and comorbidities in groups stratified according to AD and hand eczema

Overall health rating in the three groups stratified according to lifetime prevalence of AD and hand eczema is presented in Figure 1C. We found a significantly larger proportion in the group with AD and hand eczema responding, ‘less good’ or ‘poor’, compared with AD without hand eczema (*p* = 0.037). Individuals with a lifetime prevalence of hand eczema and no atopic history reported worse overall health ratings compared with those with both AD and hand eczema (*p* = 0.016). This corresponded with individuals with hand eczema alone being significantly older and reporting higher prevalences of cardiovascular comorbidities compared with those with both AD and hand eczema (Table S3).

Additionally, we examined psychiatric comorbidities in the three groups using binary logistic regression analyses—crude and adjusted ORs are presented in Table 4. We found an association between self‐reported depression, anxiety, and stress, and having both AD and hand eczema, compared with individuals with AD only. When adjusting for age, sex, educational level, alcohol, and smoking, the associations remained statistically significant for depression and anxiety. Furthermore, we found an association between self‐reported depression, anxiety, and stress, and having both AD and hand eczema, when comparing to individuals with hand eczema only. However, the associations did not remain significant after adjusting for age, sex, educational level, alcohol, and smoking, except for the association between anxiety and having both AD and hand eczema.

**TABLE 4:** Associations between psychiatric comorbidities and a lifetime prevalence of both atopic dermatitis and hand eczema.

	Included in analysis (*n*)	Crude OR [95% CI]	*p*‐Value	Included in analysis (*n*)	Model 1^a^ OR [95% CI]	*p*‐Value	Included in analysis (*n*)	Model 2^b^ OR [95% CI]	*p*‐Value
Depression									
Atopic dermatitis and hand eczema	1915	1.21 [1.02–1.43]	**0.032**	1915	1.20 [1.01–1.42]	**0.038**	1640	1.24 [1.02–1.49]	**0.027**
Atopic dermatitis, no hand eczema	1675	1		1675	1		1405	1	
Atopic dermatitis and hand eczema	1915	1.29 [1.14–1.47]	**<0.001**	1915	1.08 [0.94–1.23]	0.263	1640	1.12 [0.97–1.29]	0.137
Hand eczema, no atopic dermatitis	7166	1		7166	1		5634	1	
Anxiety									
Atopic dermatitis and hand eczema	1915	1.18 [0.98–1.42]	0.084	1915	1.24 [1.03–1.50]	**0.027**	1640	1.25 [1.02–1.54]	**0.031**
Atopic dermatitis, no hand eczema	1675	1		1675	1		1405	1	
Atopic dermatitis and hand eczema	1915	1.53 [1.32–1.77]	**<0.001**	1915	1.15 [0.99–1.34]	0.064	1640	1.19 [1.01–1.40]	**0.034**
Hand eczema, no atopic dermatitis	7166	1		7166	1		5634	1	
Stress									
Atopic dermatitis and hand eczema	1915	1.19 [1.02–1.39]	**0.031**	1915	1.16 [0.99–1.36]	0.075	1640	1.13 [0.95–1.34]	0.160
Atopic dermatitis, no hand eczema	1675	1		1675	1		1405	1	
Atopic dermatitis and hand eczema	1915	1.29 [1.15–1.46]	**<0.001**	1915	1.09 [0.96–1.23]	0.169	1640	1.13 [0.99–1.29]	0.069
Hand eczema, no atopic dermatitis	7166	1		7166	1		5634	1	

*Note*: Binary logistic regression analyses displayed as ORs with 95% CIs. Outcomes are self‐reported lifetime prevalence of physician‐diagnosed psychiatric comorbidities. Number of individuals with a self‐reported lifetime prevalence of physician‐diagnosed AD, *n* = 3590 (of these, *n* = 1915 individuals had a lifetime prevalence of hand eczema, *n* = 1675 did not). No atopic dermatitis was defined as the individuals responding ‘No’ or ‘Unknown’ to the question on a lifetime prevalence of physician‐diagnosed AD. Number of individuals with a lifetime prevalence of hand eczema, *n* = 9081 (of these, *n* = 1915 had a lifetime prevalence of atopic dermatitis, and *n* = 7166 did not). Missing answers were excluded. Data regarding missing answers can be found in Appendix S1. Bold values denote significance at the *p* < 0.05 level.

Abbreviations: AD, atopic dermatitis; CI, confidence interval; OR, odds ratio.

^a^ Adjusted for age and sex.

^b^ Adjusted for age, sex, educational level, alcohol, and smoking. Educational level was defined as lower education (unskilled or skilled profession) or higher education (higher education <3, 3–4, or >4 years). Alcohol consumption was divided into groups of ≥10 or < 10 units weekly. Smoking status was divided into daily smoking or not.

## 4 DISCUSSION

This large nationwide cross‐sectional study provided updated prevalence estimates of AD among Danish adults along with information about disease severity. AD was associated with contact sensitization and increased hand eczema prevalence. Individuals with both AD and hand eczema reported worse disease severity. Furthermore, we found an association between having both conditions and psychiatric comorbidities.

The lifetime prevalence of AD was found to be 9.0%. The 1‐year prevalence was 3.6% and the point prevalence 2.0%. Our finding of a 9.0% physician diagnosed total lifetime AD prevalence is comparable to the 9.3% reported in a recent Dutch study on the Lifelines cohort,^18^ and international findings from Europe reported by Barbarot et al. (8.3%).^2^ The point prevalence in the Lifelines cohort was 3.3%, and Barbarot et al. reported differing estimates depending on the country, for example, 2.1% in Germany and 3.6% in France.

Estimating AD prevalence is a challenging task,^3^ and several factors will have an impact on the result, for example, the age group or ethnicity of the study population, whether the study method includes clinical diagnosis or is based on a survey, and whether the study population represents the general population, outpatient clinic patients, primary care patients, and so forth. Even though clinical examination is the gold standard for diagnosing AD,^3^ this is seldom feasible in larger nationwide cross‐sectional studies. Thus, the lifetime prevalence estimate of this study was based on a modified version of a question validated by Silverberg et al.^13^ In the validation study, it was demonstrated that the question had a low sensitivity but a high specificity with regards to adult AD. Accordingly, the current study might present an underestimation of the true prevalence of AD.^19^, ^20^ With the high specificity in mind, the group of individuals with AD in this study will most likely reflect individuals who have in fact been diagnosed with AD, making characterisation of patient‐reported outcomes in the AD group more reliable.

Responders with AD were more likely to be younger and of female sex, which is in line with previous reports.^2^, ^3^, ^19^, ^20^, ^21^ In addition, the proportion of atopic comorbidities in the non‐AD and AD group were comparable to previous studies.^22^, ^23^

We found the self‐reported disease severity of AD assessed by PO‐SCORAD to be moderate to severe for 31.6% of individuals with AD. In a multinational study by Barbarot et al.,^2^ self‐reported severity of AD in a general population setting was also assessed with PO‐SCORAD. Responders with moderate to severe disease ranged from 61% to 73%. This study utilised different severity threshold scores for PO‐SCORAD (0–24, 25–49, and ≥50) compared with our study (0–27, 28–56, ≥57 as suggested by Silverberg et al.^15^), yet this is unlikely to cause as large of a difference as was observed. The difference in severity may also be a result of accessibility to and utilisation of health care that differ between countries. In Denmark, the universal health care system provides free access to health care, and thus, one could speculate whether the severity is generally scored lower in this study investigating the adult, Danish general population. Another explanation could be due to a difference in the level of selection bias between the studies.

Whether having AD increases the risk of contact sensitization compared with individuals without AD has been debated in the literature for many years. In some studies, individuals with AD demonstrated the same proportion of positive patch tests as the non‐AD group,^24^, ^25^ yet with a larger number of positive reactions to allergens.^25^ A systematic review and meta‐analysis by Hamann et al.^6^ did not find an overall association between AD and contact sensitization. However, when comparing AD patients to individuals from the general population, a positive correlation was observed (random effects pooled OR 1.54, 95% CI [1.23–1.93]).^6^ This emphasises the importance of distinguishing between a study population drawn from the general population or from a selected population referred for patch testing. In a cross‐sectional study by Diepgen et al.,^26^ a random sample from the general population of five different countries was patch tested, and 27.0% had a positive patch test result. In contrast, DeKoven et al.^27^ and Silverberg et al.^25^ have investigated patch test results in individuals referred for patch testing. In the former study, DeKoven et al. found a positive reaction in 65.4% of the patch‐tested individuals.^27^ In the latter, Silverberg et al. reported 66.5% of adults with atopic history and 65.6% of adults with no atopic history had a positive reaction.^25^ Thus, our results showed a high frequency of positive patch tests in all groups of patch‐tested individuals (overall 60.6%, AD 76.0% vs. no AD 57.0%) compared with general population studies, however not when comparing to selected patient populations. There are no validated questionnaire‐based tools for determining the proportion of positive patch tests in a general population setting. In the attempt to avoid information bias, the question regarding patch testing was accompanied by a layman description of the procedure and a photo of an individual being patch tested.^12^ One explanation for the high frequency of positive results in patch‐tested individuals could be due to selection bias to the question on ever being patch tested, if responders that previously had a positive patch test were more likely to answer this question. Nevertheless, this selection bias would likely be present in both the AD and non‐AD group. Another possible explanation for the high frequency of positive patch test results in this study could be, that too few patients in Denmark are referred for patch testing, especially individuals of male sex.

In this study, we found an association between self‐reported depression, anxiety, and stress, and having AD compared with individuals without AD that remained significant after adjusting for possible confounders such as age, sex, educational level, alcohol, and smoking. The association between AD and psychiatric comorbidities is well‐known in the literature^4^, ^28^; however, a review article from 2023 highlights the current lack of knowledge with regards to factors associated with psychiatric comorbidities in adults with AD.^29^ Two recent clinical studies suggest that lesions in the face or on hands in particular decrease quality of life.^5^, ^30^ Hence, we investigated psychiatric comorbidities among individuals with AD and hand eczema. Compared with individuals with AD and no hand eczema, there was an association with depression and anxiety that remained significant in the adjusted models. When comparing to individuals with hand eczema and no history of AD, the association was significant for crude ORs. After adjusting for age, sex, educational level, alcohol, and smoking, we only found a significant association between anxiety and having both AD and hand eczema. Considering these results, affection of the hands seems to have large impact on mental health. Surprisingly, overall health ratings were worse in the group with hand eczema and no AD compared with AD and hand eczema. Since individuals with both AD and hand eczema reported more severe hand eczema than those with hand eczema alone (Figure 1B), it is likely that the worse health ratings in the latter group were influenced by factors beyond hand eczema. Namely, individuals with hand eczema only were older, and reported higher prevalences of cardiovascular comorbidities such as hypertension, diabetes, hypercholesterolemia, and stroke, Table S3.

Anatomical distribution of eczema was previously described in adults with AD,^25^, ^31^ where one of the studies presented differences between groups stratified by disease severity of AD.^31^ To our knowledge, this is the first study mapping eczema location in groups stratified by a lifetime prevalence of AD and hand eczema. When comparing with the other groups, individuals with both AD and hand eczema were affected to a higher degree in almost every body area. We also investigated whether self‐reported disease severity differed between the groups. Individuals with AD and hand eczema reported worse hand eczema with severity assessed by the photographic guide compared with individuals with hand eczema and no atopic history. In a similar manner, individuals with AD and hand eczema reported worse AD severity assessed by PO‐SCORAD compared with individuals with AD and no hand eczema. Thus, this study demonstrated that individuals with both AD and hand eczema experience worse disease severity and involvement of more body areas.

The study has some limitations that must be considered. As with other questionnaire‐based studies, our results rely on self‐reported data. To accommodate this, validated questions were used when possible. Another limitation of the study design is that individuals with skin diseases might have a higher tendency of participating, potentially introducing selection bias. Since the analysis of non‐responders only included sex, age, and municipality group,^12^ we cannot be certain of this. We aimed at eliminating selection bias in the best way possible by stating in the invitation to the questionnaire, that all individuals were encouraged to participate regardless of having (former) eczema/rash or not.^12^

In conclusion, the lifetime prevalence of AD in the adult, Danish general population was 9.0% with nearly one third reporting moderate to severe disease. Individuals with AD reported a higher prevalence of hand eczema, and having both conditions was associated with psychiatric comorbidities and worse disease severity. This study emphasises the need for physicians and researchers to pay attention to individuals with both AD and hand eczema, as they represent a vulnerable subgroup.

## AUTHOR CONTRIBUTIONS

**Anna Schultz Vinge:** Conceptualization; formal analysis; writing—original draft; visualization; writing—review and editing; data curation. **Lone Skov:** Conceptualization; writing—review and editing; funding acquisition; supervision. **Jeanne Duus Johansen:** Conceptualization; writing—review and editing; funding acquisition; supervision. **Anna Sophie Quaade:** Conceptualization; formal analysis; writing—review and editing; funding acquisition; methodology; investigation; supervision; data curation.

## FUNDING INFORMATION

This study received funding from Kongelig Hofbuntmager Aage Bangs Foundation, the LEO Foundation grant number (LF‐ST‐21‐500002), and Copenhagen University Hospital – Herlev and Gentofte Hospital, Denmark.

## CONFLICT OF INTEREST STATEMENT

The authors have no conflicts of interest to declare.

## Supporting information


**Data S1:** Appendix.

## Data Availability

Research data are not shared.
