# Supplementary material for: Atopic dermatitis and hand eczema in Danish adults: A nationwide population‐based study
Source: Contact Dermatitis. 2024 Sep 12;92(1):21–30. doi: 10.1111/cod.14691 (PMC11669563; doi:10.1111/cod.14691)
Supplement: Supplementary file 1 — Data S1: Appendix. [file COD-92-21-s001.docx]

**Supplementary - Appendix S1**

| Supplementary table 1: Characteristics of responders according to self-reported lifetime prevalence of physician diagnosed atopic dermatitis | | | | |
| --- | --- | --- | --- | --- |
|  | **Total n=40 007** | **Atopic dermatitis n=3 601** | **No atopic dermatitis n=36 406** | ***P*-value** |
| **Age, median (IQR)** | 54.7 (25) | 42.4 (24) | 55.8 (24) | **<.001** |
| **Age, mean ± SD** | 51.9 ± 16.1 | 42.5 ± 15.1 | 52.8 ± 15.9 | **<.001** |
| **Sex, females, n (%)** | 22 680 (56.7) | 2 705 (75.1) | 19 975 (54.9) | **<.001** |
| **Comorbidities†** | | | | |
| Asthma, n (%) | 5 702 (14.3) | 1 173 (32.6) | 4 529 (12.5) | **<.001** |
| Allergic rhinitis, n (%) | 8 659 (21.7) | 1 509 (42.0) | 7 150 (19.7) | **<.001** |
| Diabetes | 2 130 (5.3) | 80 (2.2) | 2 050 (5.6) | **<.001** |
| Hypertension | 10 117 (25.3) | 574 (15.9) | 9 543 (26.2) | **<.001** |
| Hypercholesterolemia | 8 749 (21.9) | 477 (13.2) | 8 272 (22.7) | **<.001** |
| Myocardial infarction | 719 (1.8) | 19 (0.5) | 700 (1.9) | **<.001** |
| Stroke | 987 (2.5) | 45 (1.2) | 942 (2.6) | **<.001** |
| Depression | 4 978 (12.4) | 665 (18.5) | 4 313 (11.8) | **<.001** |
| Anxiety | 3 351 (8.4) | 521 (14.5) | 2 830 (7.8) | **<.001** |
| Stress | 6 434 (16.1) | 824 (22.9) | 5 610 (15.4) | **<.001** |
| **Alcohol intake >10 units weekly, n (%)** | 4 282 (11.4) | 235 (6.9) | 4 047 (11.9) | **<.001** |
| **Smoking (daily), n (%)** | 4 746 (12.1) | 392 (11.1) | 4 354 (12.2) | .057 |
| **Municipality group, n (%)** | | | | **<.001** |
| Capital | 9 707 (24.3) | 1 053 (29.2) | 8 654 (23.8) | **<.001** |
| Metropolitan | 5 499 (13.7) | 568 (15.8) | 4 931 (13.5) | **<.001** |
| Provincial | 9 540 (23.8) | 857 (23.8) | 8 683 (23.9) | .945 |
| Commuter | 6 981 (17.4) | 541 (15.0) | 6 440 (17.7) | **<.001** |
| Rural | 8 280 (20.7) | 582 (16.2) | 7 698 (21.1) | **<.001** |
| **Personal income  Grouped approximately by the deciles of equivalent disposable income from Statistics Denmark 2021‡, n (%)** | | | | 0.059 |
| 1st - 3rd deciles (lowest income)  <200 000 DKK | 8 996 (25.3) | 877 (27.0) | 8 119 (25.2) |  |
| 4th - 6th decile  200 000-300 000 DKK | 7 149 (20.1) | 652 (20.1) | 6 497 (20.1) |  |
| 7th - 10th deciles (highest income)  >300 000 DKK | 19 372 (54.5) | 1 719 (52.9) | 17 653 (54.7) |  |
| **Education** | | | | **<.001** |
| Unskilled profession | 8 130 (25.1) | 791 (25.2) | 7 339 (25.1) | .861 |
| Skilled profession | 11 005 (34.0) | 1 103 (35.2) | 9 902 (33.9) | .134 |
| Higher education < 3 years | 6 302 (19.5) | 500 (16.0) | 5 802 (19.8) | **<.001** |
| Higher education 3-4 years | 5 743 (17.7) | 675 (21.5) | 5 068 (17.3) | **<.001** |
| Higher education > 4 years | 1 187 (3.7) | 64 (2.0) | 1 123 (3.8) | **<.001** |
| **Hand eczema prevalence, n (%)** | | | | |
| Lifetime | 9 081 (22.9) | 1 915 (53.3) | 7 166 (19.8) | **<.001** |
| One year | 4 928 (12.4) | 1 206 (33.7) | 3 722 (10.3) | **<.001** |
| Point | 2 159 (5.5) | 610 (17.1) | 1 549 (4.3) | **<.001** |
| **Severity of hand eczema (photographic guide)**  **Averagely the past year, n (%)** | | | | **<.001** |
| Mild | 3 168 (64.9) | 728 (60.8) | 2 440 (66.3) | **<.001** |
| Moderate to very severe | 1 710 (35.1) | 469 (39.2) | 1 241 (33.7) | **<.001** |
| **Severity of hand eczema VAS (range 0-10)  Averagely the past year, median (IQR)** | 2.7 (2.7) | 3.0 (3.0) | 2.6 (2.4) | **<.001** |
| **Severity of hand eczema VAS (range 0-10)  Averagely the past year, mean ± SD** | 3.3 ± 2.0 | 3.6 ± 2.1 | 3.2 ± 2.0 | **<.001** |
| **Severity of atopic dermatitis (PO-SCORAD)§**  **Currently, n (%)** | | | |  |
| Mild | N/A | 951 (68.4) | N/A |  |
| Moderate to severe | N/A | 439 (31.6) | N/A |  |
| **Sick leave the past year > 1 week (any reason), n (%)** | 7 897 (21.5) | 917 (26.9) | 6 980 (20.9) | **<.001** |
| **Duration of total sick leave the past year (weeks), median (IQR)** | 3.0 (7.7) | 3.0 (7.7) | 3.0 (7.7) | .796 |
| **Duration of total sick leave the past year (weeks), mean ± SD** | 9.6 ± 14.9 | 9.6 ± 14.8 | 9.6 ± 14.9 | .996 |
| **GP consultations past year (any reason), median (IQR)** | 2.0 (3) | 2.0 (3) | 2.0 (2) | **<.001** |
| **GP consultations past year (any reason), mean ± SD** | 2.7 ± 3.3 | 3.2 ± 3.9 | 2.6 ± 3.2 | **<.001** |
| **Ever patch tested, n (%)** | 7 375 (18.7) | 1 398 (39.5) | 5 977 (16.7) | **<.001** |
| **Positive patch test  Of patch tested individuals, n (%)** | 4 101 (60.6) | 989 (76.0) | 3 112 (57.0) | **<.001** |
| **Number of positive allergens from patch test ≥2  Of patch tested individuals, n (%)** | 1 033 (15.3) | 343 (26.4) | 690 (12.6) | **<.001** |
| **Positive allergen   Of patch tested individuals, n (%)** | | | | |
| Nickel | 1 337 (19.8) | 360 (27.7) | 977 (17.9) | **<.001** |
| Chrome | 231 (3.4) | 74 (5.7) | 157 (2.9) | **<.001** |
| Cobalt | 177 (2.6) | 58 (4.5) | 119 (2.2) | **<.001** |
| Perfume | 816 (12.1) | 263 (20.2) | 553 (10.1) | **<.001** |
| Colorants | 284 (4.2) | 120 (9.2) | 164 (3.0) | **<.001** |
| Preservatives | 384 (5.7) | 120 (9.2) | 264 (4.8) | **<.001** |
| Acrylates | 31 (0.5) | 8 (0.6) | 23 (0.4) | .353 |
| Epoxy | 64 (0.9) | 15 (1.2) | 49 (0.9) | .392 |
| Rubber chemicals | 236 (3.5) | 82 (6.3) | 154 (2.8) | **<.001** |
| Colophonium | 72 (1.1) | 19 (1.5) | 53 (1.0) | .122 |
| Others/do not remember which allergy | 2 038 (30.1) | 573 (44.0) | 1 465 (26.8) | **<.001** |
| Note: For numeric variables, differences between group medians were calculated using Mann-Whitney U test and between group means using students t-test. For categorical variables, differences between group proportions were calculated using Chi-square test. If an overall significant difference was found, post hoc testing with pairwise Z-tests based on adjusted standardized residuals was performed. Missing answers were excluded. For data regarding **missing answers**, see below. Bold values denote significance at the *P*<.05 level. Abbreviations: DKK, Danish kroner; GP, general practitioner; IQR, interquartile range; PO-SCORAD, Patient‐Oriented SCORing for AD; SD, standard deviation; VAS, visual analogue scale.  † Self-reported lifetime prevalence of physician diagnosed cardiovascular and psychiatric comorbidities.  ‡ Equivalent disposable income is a measure published by Statistics Denmark every year that allows comparison of disposable income on an individual level across Danish households. It is calculated based on household income and adjusted for number of family members. It is divided into 10 decile groups - for the year 2021: 1st decile 95 473 DKK, 2nd 166 971 DKK, 3rd 196 935 DKK, 4th 227 383 DKK, 5th 259 036 DKK, 6th 291 453 DKK, 7th 327 257 DKK, 8th 371 556 DKK, 9th 437 776 DKK, and 10th 779 827 DKK. The income groups in the questionnaire were designed before these decile limits were calculated, and as a consequence hereof, the groups stated above is an attempt to approximate these decile limits as well as possible.  § 1 436 individuals reported a one year prevalence of atopic dermatitis and were asked to self-report the severity of atopic dermatitis as assessed by the PO-SCORAD. ≤27: Mild disease. >27: Moderate to severe disease. | | | | |

| Supplementary table 2: Associations between cardiovascular or psychiatric comorbidities and atopic dermatitis | | | | | | | | | | | | | | | | |
| --- | --- | --- | --- | --- | --- | --- | --- | --- | --- | --- | --- | --- | --- | --- | --- | --- |
|  | **Included in analysis (n)** | **Crude OR (95% CI)** | | ***P*-value** | **Included in analysis (n)** | | **Model 1† OR (95% CI)** | | | ***P*-value** | **Included in analysis (n)** | | **Model 2‡ OR (95 % CI)** | | ***P*-value** | |
| **Diabetes** | | | | | | | | | | | | | | | | |
| Atopic dermatitis | 3 601 | 0.38 (0.30-0.48) | | **<.001** | 3 601 | | 0.65 (0.51-0.81) | | | **<.001** | 3 050 | | 0.68 (0.53-0.87) | | **.003** | |
| No atopic dermatitis | 36 406 | 1 | |  | 36 406 | | 1 | | |  | 27 901 | | 1 | |  | |
| **Hypertension** | | | | | | | | | | | | | | | | |
| Atopic dermatitis | 3 601 | 0.53 (0.49-0.59) | | **<.001** | 3 601 | | 0.99 (0.90-1.09) | | | .833 | 3 050 | | 1.01 (0.91-1.13) | | .841 | |
| No atopic dermatitis | 36 406 | 1 | |  | 36 406 | | 1 | | |  | 27 901 | | 1 | |  | |
| **Hypercholesterolemia** | | | | | | | | | | | | | | | | |
| Atopic dermatitis | 3 601 | 0.52 (0.47-0.57) | | **<.001** | 3 601 | | 0.92 (0.83-1.03) | | | **.**134 | 3 050 | | 0.91 (0.80-1.02) | | .102 | |
| No atopic dermatitis | 36 406 | 1 | |  | 36 406 | | 1 | | |  | 27 901 | | 1 | |  | |
| **Myocardial infarction** | | | | | | | | | | | | | | | | |
| Atopic dermatitis | 3 601 | 0.27 (0.17-0.43) | | **<.001** | 3 601 | | 0.63 (0.40-1.00) | | | **.049** | 3 050 | | 0.63 (0.38-1.07) | | .086 | |
| No atopic dermatitis | 36 406 | 1 | |  | 36 406 | | 1 | | |  | 27 901 | | 1 | |  | |
| **Stroke** | | | | | | | | | | | | | | | | |
| Atopic dermatitis | 3 601 | 0.48 (0.35-0.64) | | **<.001** | 3 601 | | 0.89 (0.65-1.21) | | | .450 | 3 050 | | 0.97 (0.70-1.35) | | .867 | |
| No atopic dermatitis | 36 406 | 1 | |  | 36 406 | | 1 | | |  | 27 901 | | 1 | |  | |
| **Depression** | | | | | | | | | | | | | | | | |
| Atopic dermatitis | 3 601 | 1.69 (1.54-1.84) | | **<.001** | 3 601 | | 1.35 (1.23-1.48) | | | **<.001** | 3 050 | | 1.33 (1.20-1.46) | | **<.001** | |
| No atopic dermatitis | 36 406 | 1 | |  | 36 406 | | 1 | | |  | 27 901 | | 1 | |  | |
| **Anxiety** | | | | | | | | | | | | | | | | |
| Atopic dermatitis | 3 601 | 2.01 (1.82-2.22) | | **<.001** | 3 601 | | 1.35 (1.22-1.50) | | | **<.001** | 3 050 | | 1.36 (1.21-1.52) | | **<.001** | |
| No atopic dermatitis | 36 406 | 1 | |  | 36 406 | | 1 | | |  | 27 901 | | 1 | |  | |
| **Stress** | | | | | | | | | | | | | | | | |
| Atopic dermatitis | 3 601 | 1.63 (1.50-1.77) | | **<.001** | 3 601 | | 1.32 (1.22-1.44) | | | **<.001** | 3 050 | | 1.34 (1.22-1.47) | | **<.001** | |
| No atopic dermatitis | 36 406 | 1 | |  | 36 406 | | 1 | | |  | 27 901 | | 1 | |  | |
| Note: Binary logistic regression analyses displayed as ORs with 95 % CIs. Outcomes are self-reported physician lifetime prevalence of the mentioned cardiovascular and psychiatric comorbidities. Missing answers were excluded. For data regarding **missing answers,** see below. Bold values denote significance at the *P*<.05 level. Abbreviations: CI, confidence interval; OR, odds ratio.  † Adjusted for age and sex.  ‡ Adjusted for age, sex, educational level, alcohol, and smoking. Educational level was defined as lower education (unskilled or skilled profession) or higher education (higher education <3 years, 3-4 years, or >4 years). Alcohol consumption was divided into groups of ≥10 units weekly or <10 units. Smoking status was divided into daily smoking or not. | | | | | | | | | | | | | | | | |
| Supplementary table 3: Self-reported overall health, medical attention seeking behaviour, sick leave, severity of atopic dermatitis, patch testing, socioeconomic status, and comorbidities in individuals with and without atopic dermatitis including with and without hand eczema. | | | | | | | | | | | | | | | |  |
|  | | **Total n=39 701** | **Atopic dermatitis n=3 590** | | | | | **P-value  AD+HE vs. AD no HE** | **No atopic dermatitis n=36 111** | | | | | **P-value  HE no AD vs. no AD no HE** | |  |
|  |  |  | **Hand eczema n=1 915** | | | **No hand eczema n=1 675** | |  | **Hand eczema n= 7 166** | | | **No hand eczema n=28 945** | |  |  |  |
| **Age, median (IQR)** | | 54.6 (24.8) | 43.8 (24.1) | | | 39.9 (24.3) | | **<.001** | 53.9 (23.1) | | | 56.1 (23.6) | | **<.001** | |  |
| **Sex, females, n (%)** | | 22 541 (56.8) | 1 481 (77.3) | | | 1 220 (72.8) | | **.002** | 4 608 (64.3) | | | 15 232 (52.6) | | **<.001** | |  |
| **Overall health rating** | |  |  | | |  | | **.039** |  | | |  | | **<.001** | |  |
| Excellent | | 4 625 (11.9) | 198 (10.5) | | | 212 (12.9) | | **.028** | 694 (9.9) | | | 3 521 (12.4) | | **<.001** | |  |
| Very good | | 14 992 (38.4) | 732 (39.0) | | | 671 (40.9) | | .243 | 2 480 (35.2) | | | 11 109 (39.0) | | **<.001** | |  |
| Good | | 14 408 (36.9) | 676 (36.0) | | | 559 (34.1) | | .236 | 2 677 (38.0) | | | 10 496 (36.9) | | .072 | |  |
| Less good | | 4 215 (10.8) | 231 (12.3) | | | 172 (10.5) | | .092 | 988 (14.0) | | | 2 824 (9.9) | | **<.001** | |  |
| Poor | | 767 (2.0) | 42 (2.2) | | | 27 (1.6) | | .208 | 197 (2.8) | | | 501 (1.8) | | **<.001** | |  |
| **GP consultations past year (any reason), median (IQR)** | | 2.0 (3) | 2.0 (3) | | | 2.0 (3) | | .075 | 2.0 (3) | | | 2.0 (2) | | **<.001** | |  |
| **GP consultations past year (any reason), mean ± SD** | |  | 3.3 ± 4.1 | | | 3.1 ± 3.7 | | .084 | 2.9 ± 3.5 | | | 2.5 ± 3.2 | | **<.001** | |  |
| **Sick leave the past year > 1 week (any reason), n (%)** | | 7 856 (21.5) | 494 (27.3) | | | 422 (26.5) | | .586 | 1 651 (25.4) | | | 5 289 (19.9) | | **<.001** | |  |
| **Duration of total sick leave the past year (weeks), median (IQR)** | | 3.0 (7.7) | 3.0 (7.7) | | | 2.5 (6.7) | | .904 | 3.0 (11.0) | | | 3.0 (7.7) | | **<.001** | |  |
| **Duration of total sick leave the past year (weeks), mean ± SD** | | 9.6 ± 14.9 | 9.7 ± 14.8 | | | 9.4 ± 14.6 | | .702 | 10.6 ± 15.6 | | | 9.3 ± 14.7 | | **.003** | |  |
| **Severity of atopic dermatitis (PO-SCORAD) currently, n (%)  Of individuals with a one-year prevalence of atopic dermatitis**† | | | | | | | | **.004** | **N/A** | | | **N/A** | |  | |  |
| Mild (≤27) | | 949 (68.4) | 580 (65.7) | | | 369 (73.1) | | **.004** |  | | |  | |  | |  |
| Moderate-severe (>27) | | 439 (31.6) | 303 (34.3) | | | 136 (26.9) | | **.004** |  | | |  | |  | |  |
| **Severity of atopic dermatitis (PO-SCORAD) currently, n (%)  Of individuals with a point prevalence of atopic dermatitis**‡ | | | | | | | | .411 | **N/A** | | | **N/A** | |  | |  |
| Mild (≤27) | | 421 (55.0) | 282 (54.0) | | | 139 (57.2) | |  |  | | |  | |  | |  |
| Moderate-severe (>27) | | 344 (45.0) | 240 (46.0) | | | 104 (42.8) | |  |  | | |  | |  | |  |
| **Ever patch tested, n (%)** | | 7 330 (18.7) | 956 (50.7) | | | 439 (26.7) | | **<.001** | 2 233 (31.7) | | | 3 702 (13.0) | | **<.001** | |  |
| **Positive patch test (of patch tested individuals), n (%)** | | 4 084 (60.8) | 690 (77.3) | | | 297 (73.2) | | .108 | 1 353 (67.5) | | | 1 744 (51.0) | | **<.001** | |  |
| **Annual household income < 600,000 DKK, n (%)** | | 17 652 (54.9) | 829 (52.1) | | | 684 (50.4) | | .368 | 3 257 (56.5) | | | 12 882 (55.0) | | **.040** | |  |
| **Personal income Grouped approximately by the deciles of equivalent disposable income from Stastistics Denmark 2021, n (%)** | | | | | | | | **.003** |  | | |  | | **.016** | |  |
| 1st - 3rd deciles (lowest income)  <200,000 DKK | | 8 912 (25.3) | 423 (24.5) | | | 452 (29.8) | | **.001** | 1 599 (25.4) | | | 6 438 (25.0) | | .548 | |  |
| 4th - 6th decile  200,000-300,000 DKK | | 7 094 (20.1) | 361 (20.9) | | | 288 (19.0) | | .168 | 1 339 (21.3) | | | 5 106 (19.8) | | **.012** | |  |
| 7th - 10th deciles (highest income)  >300,000 DKK | | 19 255 (54.6) | 940 (54.5) | | | 776 (51.2) | | .058 | 3 359 (53.3) | | | 14 180 (55.1) | | **.011** | |  |
| **Education** | |  |  | | |  | | **<.001** |  | | |  | | **<.001** | |  |
| Unskilled profession | | 8 063 (25.1) | 363 (21.6) | | | 425 (29.4) | | **<.001** | 1 349 (23.1) | | | 5 926 (25.6) | | **<.001** | |  |
| Skilled profession | | 10 932 (34.0) | 615 (36.5) | | | 487 (33.7) | | .097 | 2 070 (35.4) | | | 7 760 (33.5) | | **.006** | |  |
| Higher education < 3 years | | 6 256 (19.5) | 303 (18.0) | | | 197 (13.6) | | **.001** | 1 223 (20.9) | | | 4 533 (19.6) | | **.021** | |  |
| Higher education 3-4 years | | 5 720 (17.8) | 374 (22.2) | | | 300 (20.8) | | .322 | 995 (17.0) | | | 4 051 (17.5) | | .394 | |  |
| Higher education > 4 years | | 1 171 (3.6) | 28 (1.7) | | | 36 (2.5) | | .103 | 211 (3.6) | | | 896 (3.9) | | .354 | |  |
| **Municipality group, n (%)** | |  |  | | |  | | .800 |  | | |  | | .186 | |  |
| Capital | | 9 645 (24.3) | 558 (29.1) | | | 493 (29.4) | |  | 1 761 (24.6) | | | 6 833 (23.6) | |  | |  |
| Metropolitan | | 5 469 (13.8) | 295 (15.4) | | | 272 (16.2) | |  | 961 (13.4) | | | 3 941 (13.6) | |  | |  |
| Provincial | | 9 459 (23.8) | 449 (23.4) | | | 405 (24.2) | |  | 1 684 (23.5) | | | 6 921 (23.9) | |  | |  |
| Commuter | | 6 931 (17.5) | 294 (15.4) | | | 245 (14.6) | |  | 1 301 (18.2) | | | 5 091 (17.6) | |  | |  |
| Rural | | 8 197 (20.6) | 319 (16.7) | | | 260 (15.5) | |  | 1 459 (20.4) | | | 6 159 (21.3) | |  | |  |
| **Comorbidities**§ | |  |  | | |  | |  |  | | |  | |  | |  |
| Diabetes | | 2 110 (5.3) | 42 (2.2) | | | 38 (2.3) | | .879 | 413 (5.8) | | | 1 617 (5.6) | | .561 | |  |
| Hypertension | | 10 024 (25.2) | 336 (17.5) | | | 237 (14.1) | | **.006** | 1 776 (24.8) | | | 7 675 (26.5) | | **.003** | |  |
| Hypercholesterolemia | | 8 687 (21.9) | 277 (14.5) | | | 199 (11.9) | | **.023** | 1 570 (21.9) | | | 6 641 (22.9) | | .061 | |  |
| AMI | | 707 (1.8) | 12 (0.6) | | | 7 (0.4) | | .390 | 138 (1.9) | | | 550 (1.9) | | .887 | |  |
| Stroke | | 977 (2.5) | 29 (1.5) | | | 16 (1.0) | | .133 | 184 (2.6) | | | 748 (2.6) | | .937 | |  |
| Depression | | 4 944 (12.5) | 378 (19.7) | | | 284 (17.0) | | **.032** | 1 146 (16.0) | | | 3 136 (10.8) | | **<.001** | |  |
| Anxiety | | 3 331 (8.4) | 295 (15.4) | | | 224 (13.4) | | .084 | 763 (10.6) | | | 2 049 (7.1) | | **<.001** | |  |
| Stress | | 6 401 (16.1) | 465 (24.3) | | | 356 (21.3) | | **.031** | 1 425 (19.9) | | | 4 155 (14.4) | | **<.001** | |  |
| Note: For numeric variables, differences between group medians were calculated using Mann-Whitney U test (2 groups) and Kruskal-Wallis test (>2 groups), and between group means using student's t-test (2 groups) or ANOVA (>2 groups). For categorical variables, differences between group proportions was calculated using Chi-square test. If an overall significant difference was found, post hoc testing with pairwise Z-tests based on adjusted standardized residuals was performed. Bold values denote significance at the *P*<.05 level.  † 1 431 individuals had AD within the past year. Of these, 914 had a lifetime prevalence of HE and 517 did not  ‡ 790 individuals had current AD. Of these, 543 had a lifetime prevalence of HE and 247 did not  § Selfreported physician lifetime prevalence of the mentioned cardiovascular and psychiatric comorbidities. | | | | | | | | | | | | | | | |  |

| Supplementary table 4a: Distribution of current eczema in different anatomical sites for individuals with atopic dermatitis and hand eczema (n=1 914) and individuals with hand eczema and no atopic dermatitis (n=7 158). | | | |
| --- | --- | --- | --- |
|  | **Atopic dermatitis and hand eczema n=1 914** | **Hand eczema,  no atopic dermatitis n=7 158** | ***P*-value** |
| **Localization of current eczema, n (%)** | | | |
| Face and ears | 279 (16.1) | 600 (9.8) | **<.001** |
| Scalp | 224 (13.4) | 567 (9.4) | **<.001** |
| Neck and upper chest | 223 (13.2) | 338 (5.7) | **<.001** |
| Armpit | 66 (4.0) | 134 (2.3) | **<.001** |
| Arms | 341 (19.2) | 638 (10.3) | **<.001** |
| Hands | 609 (32.1) | 1 547 (21.9) | **<.001** |
| Abdomen | 88 (5.4) | 236 (4.0) | **.016** |
| Back | 135 (8.2) | 264 (4.5) | **<.001** |
| Inguinal area | 78 (4.7) | 229 (3.9) | .134 |
| Buttocks | 85 (5.2) | 223 (3.8) | **.012** |
| Genitals | 50 (3.1) | 167 (2.9) | .617 |
| Legs | 257 (14.9) | 582 (9.6) | **<.001** |
| Feet | 152 (9.2) | 488 (8.2) | .194 |
| Note: Differences between group proportions were calculated using Chi-square test. Missing answers were excluded. For data regarding **missing answers,** see below. Bold values denote significance at the *P*<.05 level. | | | |

| Supplementary table 4b: Distribution of current eczema in different anatomical sites for individuals with atopic dermatitis and hand eczema (n=1 914) and individuals with atopic dermatitis without hand eczema (n=1 671). | | | |
| --- | --- | --- | --- |
|  | **Atopic dermatitis and hand eczema n=1 914** | **Atopic dermatitis,**  **no hand eczema**  **n=1 671** | ***P*-value** |
| **Localization of current eczema, n (%)** | | | |
| Face and ears | 279 (16.1) | 178 (12.0) | **.001** |
| Scalp | 224 (13.4) | 145 (10.0) | **.004** |
| Neck and upper chest | 223 (13.2) | 91 (6.4) | **<.001** |
| Armpit | 66 (4.0) | 32 (2.3) | **.007** |
| Arms | 341 (19.2) | 146 (9.5) | **<.001** |
| Hands | 609 (32.1) | 0 (0.0) | **<.001** |
| Abdomen | 88 (5.4) | 56 (4.0) | .075 |
| Back | 135 (8.2) | 63 (4.5) | **<.001** |
| Inguinal area | 78 (4.7) | 38 (2.7) | **.004** |
| Buttocks | 85 (5.2) | 32 (2.3) | **<.001** |
| Genitals | 50 (3.1) | 28 (2.0) | .066 |
| Legs | 257 (14.9) | 101 (6.8) | **<.001** |
| Feet | 152 (9.2) | 46 (3.3) | **<.001** |
| Note: Differences between group proportions were calculated using Chi-square test. Missing answers were excluded. For data regarding **missing answers,** see below. Bold values denote significance at the *P*<.05 level. | | | |

**Missing data**

| Missing data for Table 1 and Supplementary table 1 | | | |
| --- | --- | --- | --- |
|  | **Branching** | **Answers** | **Missing answers†,  total (atopic dermatitis, no atopic dermatitis‡)** |
| **Age** |  | 40 007 | 0§ |
| **Sex** |  | 40 007 | 0§ |
| **Asthma** |  | 39 909 | 98 (AD 3, no AD 95) |
| **Allergic rhinitis** |  | 39 864 | 143 (AD 9, no AD 134) |
| **Cardiovascular and psychiatric comorbidities** |  | 40 007 | 0¶ |
| **Alcohol intake >10 units weekly** |  | 37 560 | 2 447 (AD 175, no AD 2 272) |
| **Smoking (daily)** |  | 39 110 | 897 (AD 81, no AD 816) |
| **Municipality group** |  | 40 007 | 0§ |
| **Personal income** |  | 35 517 | 4 490 (AD 353, no AD 4 137) **††** |
| **Education** |  | 32 367 | 7 640 (AD 468, no AD 7 172) **‡‡** |
| **Lifetime prevalence of HE** |  | 39 701 | 306 (AD 11, no AD 295) |
| **1-year prevalence of HE** | If "Yes" to lifetime prevalence of HE (9 081) | 9 007 | 74 (AD 10, no AD 64) |
| **Point prevalence of HE** | If "Yes" to 1-year prevalence of HE (4 928) | 4 875 | 53 (AD 8, no AD 45) |
| **Severity of hand eczema (photoguide) averagely the past year** | If "Yes" to 1-year prevalence of HE (4 928) | 4 878 | 50 (AD 9, no AD 41) |
| **Severity of hand eczema (VAS) averagely the past year** | If "Yes" to 1-year prevalence of HE (4 928) | 4 769 | 159 (AD 34, no AD 125) |
| **Severity of atopic dermatitis (PO-SCORAD) currently** | If "Yes, currently" or "Not currently, but within the past year" to the question regarding having AD still (1 436) | 1 390 | 46 (AD 46, no AD 0) |
| **Sick leave the past year > 1 week (any reason)** |  | 36 766 | 3 241 (AD 194, no AD 3 047) |
| **Duration of total sick leave the past year (weeks)** | If "Yes" to sick leave (any reason) (16 213) | 16 213 | 0 |
| **GP consultations past year (any reason)** |  | 39 100 | 907 (AD 78, no AD 829) |
| **Ever patch tested** |  | 39 395 | 612 (AD 65, no AD 547) |
| **Positive patch test (of patch tested individuals)** | If "Yes" to ever patch tested (7 375) | 6 762 | 613 (AD 97, no AD 516) ‡‡ |
| **Number of positive allergens from patch test ≥2** | If "Yes" to ever patch tested (7 375) | 6 762 | 613 (AD 97, no AD 516) ‡‡ |
| **Positive allergen** | If "Yes" to ever patch tested (7 375) | 6 762 | 613 (AD 97, no AD 516) §§ |
| Abbreviations: AD, atopic dermatitis; GP, general practitioner; HE, hand eczema; PO-SCORAD, Patient‐Oriented SCORing for AD; VAS, visual analogue scale. † Of the 40 007 individuals responding to the question on lifetime prevalence of physician diagnosed AD.  ‡ No atopic dermatitis was defined as the individuals responding "No" or "Unknown" to the question on lifetime prevalence of physician diagnosed AD.  § Information on age, sex and municipality group was retrieved from the civil registration system, and thus, there were no missing answers.  ¶ The question was phrased as a checklist, which means the respondents only answered "Yes" to the diseases, and as a consequence, "No" was not registered. We assigned "No" to the individuals from the study population (40 007) who did not respond "Yes" to each comorbidity. Hence, there were no missing answers.  **††** Of these missing answers, 2 737 individuals (AD 223, no AD 2 514) responded that they did not wish to disclose their annual personal income. **‡‡** Individuals responding "unknown" were excluded as missing answers.  §§ Individuals responding "unknown" were excluded as missing answers. The question was phrased as a checklist, which means the respondents only answered "Yes" to positive reactions to different allergens, and as a consequence, "No" was not registered. We assigned "No" to the individuals with a positive patch test who did not respond "Yes" to the concerned allergen. | | | |

| Missing data for Table 2 | | |
| --- | --- | --- |
|  | **Included in analysis, n Total (AD, no AD)** | **Missing answers†, n Total (AD, no AD)** |
| **Positive patch test (of patch tested individuals)** | | |
| Crude | 6 762 (AD 1 301, no AD 5 461) | 613 (AD 97, no AD 516)‡ |
| Model 1 | 6 762 (AD 1 301, no AD 5 461) | 613 (AD 97, no AD 516)‡ |
| Model 2 | 5 501 (AD 1 144, no AD 4 357) | 1874 (AD 254, no AD 1620)‡ |
| **≥2 positive allergens from patch test (of patch tested individuals)** | | |
| Crude | 6 762 (AD 1 301, no AD 5 461) | 613 (AD 97, no AD 516)‡ |
| Model 1 | 6 762 (AD 1 301, no AD 5 461) | 613 (AD 97, no AD 516)‡ |
| Model 2 | 5 501 (AD 1 144, no AD 4 357) | 1 874 (AD 254, no AD 1 620)‡ |
| Abbreviations: AD, atopic dermatitis.  † Of the 7 375 individuals responding both to the question on a lifetime prevalence of physician diagnosed AD and responding "Yes" to ever being patch tested (n=1 398 with AD and n=5 977 without). Information on age and sex was retrieved from the civil registration system, and thus, there were no missing answers regarding this data.  ‡ Individuals responding "Unknown" to result of patch test were excluded as missing answers. | | |
| Missing data for Table 3 | | |
|  | **Included in analysis, n Total (lower educational lever, higher educational level)** | **Missing answers†, n Total (lower educational lever, higher educational level)** |
| **Lifetime prevalence of hand eczema** | | |
| Crude | 3 128 (lower educational level 1 890, higher educational level 1 238) | 5 (lower educational level 4, higher educational level 1) |
| Model 1 | 3 128 (lower educational level 1 890, higher educational level 1 238) | 5 (lower educational level 4, higher educational level 1) |
| Model 2 | 3 124 (lower educational level 1 888, higher educational level 1 236) | 9 (lower educational level 6, higher educational level 3) |
| † Of the 3 133 individuals reporting a lifetime prevalence of physician diagnosed AD and responding to both the question on a lifetime prevalence of hand eczema and educational level were included in analysis (n=1 894 with lower educational level and n=1 239 with higher educational level). Individuals responding "Unknown" to educational level were excluded as missing answers. Information on age and sex was retrieved from the civil registration system, and thus, there were no missing answers regarding this data. | | |

| Missing data for Table 4 | | |
| --- | --- | --- |
|  | **Included in analysis, n Total AD (HE; no HE) Total HE (AD; no AD)** | **Missing answers†, n** |
| **Depression** |  |  |
| Crude | 3 590 (1 915; 1 675) 9 081 (1 915; 7 166) | 0 |
| Model 1 | 3 590 (1 915; 1 675) 9 081 (1 915; 7 166) | 0 |
| Model 2 | 3 045 (1 640; 1 405) 7 274 (1 640; 5 634) | 545 (275; 270)  1 807 (275; 1 532) |
| **Anxiety** |  |  |
| Crude | 3 590 (1 915; 1 675) 9 081 (1 915; 7 166) | 0 |
| Model 1 | 3 590 (1 915; 1 675) 9 081 (1 915; 7 166) | 0 |
| Model 2 | 3 045 (1 640; 1 405) 7 274 (1 640; 5 634) | 545 (275; 270)  1 807 (275; 1 532) |
| **Stress** |  |  |
| Crude | 3 590 (1 915; 1 675) 9 081 (1 915; 7 166) | 0 |
| Model 1 | 3 590 (1 915; 1 675) 9 081 (1 915; 7 166) | 0 |
| Model 2 | 3 045 (1 640; 1 405) 7 274 (1 640; 5 634) | 545 (275; 270)  1 807 (275; 1 532) |
| Abbreviations: AD, atopic dermatitis; HE, hand eczema. † Of the individuals with AD and HE (n=1 915), AD and no HE (n=1 675), and HE no AD (n=7 166). The question on comorbidities was phrased as a checklist, which means the respondents only answered "Yes" to the diseases, and as a consequence, "No" was not registered. We assigned "No" to the individuals from the study population who did not respond "Yes" to each comorbidity. Hence, there were no missing answers for psychiatric comorbidities. Further, information on age and sex was retrieved from the civil registration system, and thus, there were no missing answers regarding this data. | | |

| Missing data for Supplementary table 2 | | |
| --- | --- | --- |
|  | **Included in analysis, n Total (AD, no AD)** | **Missing answers†, n Total (AD, no AD)** |
| **Diabetes** |  |  |
| Crude | 40 007 (3 601, 36 406) | 0 |
| Model 1 | 40 007 (3 601, 36 406) | 0 |
| Model 2 | 30 951 (3 050, 27 901) | 9 056 (551, 8 505) |
| **Hypertension** |  |  |
| Crude | 40 007 (3 601, 36 406) | 0 |
| Model 1 | 40 007 (3 601, 36 406) | 0 |
| Model 2 | 30 951 (3 050, 27 901) | 9 056 (551, 8 505) |
| **Hypercholesterolemia** |  |  |
| Crude | 40 007 (3 601, 36 406) | 0 |
| Model 1 | 40 007 (3 601, 36 406) | 0 |
| Model 2 | 30 951 (3 050, 27 901) | 9 056 (551, 8 505) |
| **Myocardial infarction** |  |  |
| Crude | 40 007 (3 601, 36 406) | 0 |
| Model 1 | 40 007 (3 601, 36 406) | 0 |
| Model 2 | 30 951 (3 050, 27 901) | 9 056 (551, 8 505) |
| **Stroke** |  |  |
| Crude | 40 007 (3 601, 36 406) | 0 |
| Model 1 | 40 007 (3 601, 36 406) | 0 |
| Model 2 | 30 951 (3 050, 27 901) | 9 056 (551, 8 505) |
| **Depression** |  |  |
| Crude | 40 007 (3 601, 36 406) | 0 |
| Model 1 | 40 007 (3 601, 36 406) | 0 |
| Model 2 | 30 951 (3 050, 27 901) | 9 056 (551, 8 505) |
| **Anxiety** |  |  |
| Crude | 40 007 (3 601, 36 406) | 0 |
| Model 1 | 40 007 (3 601, 36 406) | 0 |
| Model 2 | 30 951 (3 050, 27 901) | 9 056 (551, 8 505) |
| **Stress** |  |  |
| Crude | 40 007 (3 601, 36 406) | 0 |
| Model 1 | 40 007 (3 601, 36 406) | 0 |
| Model 2 | 30 951 (3 050, 27 901) | 9 056 (551, 8 505) |
| Abbreviations: AD, atopic dermatitis. † The question on comorbidities was phrased as a checklist, which means the respondents only answered "Yes" to the diseases, and as a consequence, "No" was not registered. We assigned "No" to the individuals from the study population (40 007) who did not respond "Yes" to each comorbidity. Hence, there were no missing answers regarding this information. | | |

| Missing data for Supplementary table 3 | | | |
| --- | --- | --- | --- |
|  | **Branching** | **Answers** | **Missing answers†,  total (AD and HE, AD no HE, HE no AD, no AD or HE‡)** |
| **Age** |  | 39 701 | 0§ |
| **Sex** |  | 39 701 | 0§ |
| **Overall health rating** |  | 39 007 | 694 (AD and HE 36, AD np HE 34, HE no AD 130, no AD or HE 494) |
| **GP consultations past year (any reason)** |  | 38 822 | 879 (AD and HE 37, AD no HE 39, HE no AD 160, no AD or HE 643) |
| **Sick leave the past year > 1 week (any reason)** |  | 36 531 | 3 170 (AD and HE 108, AD no HE 83, HE no AD 673, no AD or HE 2 306) |
| **Duration of total sick leave the past year (weeks)** | If "Yes" to sick leave > 1 week (any reason) (7 856) | 7 856 | 0 |
| **Severity of atopic dermatitis (PO-SCORAD) currently** | If "Yes, currently" or "Not currently, but within the past year" to the question regarding having AD still (1 431) | 1 388 | 43 (AD and HE 31, AD no HE 12) |
| **Severity of atopic dermatitis (PO-SCORAD) currently** | If "Yes, currently" to the question regarding having AD still (790) | 765 | 25 (AD and HE 21, AD no HE 4) |
| **Ever patch tested** |  | 39 103 | 598 (AD and HE 31, AD no HE 31, HE no AD 118, no AD or HE 418) |
| **Positive patch test (of patch tested individuals)** | If "Yes" to ever patch tested (7 330) | 6 722 | 608 (AD and HE 63, AD no HE 33, HE no AD 228, no AD or HE 284) ‡‡ |
| **Annual household income** |  | 32 137 | 7 564 (AD and HE 324, AD no HE 319, HE no AD 1 401, no AD or HE 5 520)**††** |
| **Personal income** |  | 35 261 | 4 440 (AD and HE 191, AD no HE 159, HE no AD 869, no AD or HE 3 221) §§ |
| **Education** |  | 32 142 | 7 559 (AD and HE 232, AD no HE 230, HE no AD 1 318, no AD or HE 5 779)‡‡ |
| **Municipality group** |  | 39 701 | 0§ |
| **Cardiovascular and psychiatric comorbidities** |  | 39 701 | 0¶ |
| Abbreviations: AD, atopic dermatitis; GP, general practitioner; HE, hand eczema; PO-SCORAD, Patient‐Oriented SCORing for AD. † Of the 39 701 individuals responding to the question on lifetime prevalence of physician diagnosed AD and lifetime prevalence of hand eczema.  ‡ No atopic dermatitis was defined as the individuals responding "No" or "Unknown" to the question on lifetime prevalence of physician diagnosed AD.  § Information on age, sex and municipality group was retrieved from the civil registration system, and thus, there were no missing answers.  ¶ The question was phrased as a checklist, which means the respondents only answered "Yes" to the diseases, and as a consequence, "No" was not registered. We assigned "No" to the individuals from the study population (39 701) who did not respond "Yes" to each comorbidity. Hence, there were no missing answers.  **††** Of these missing answers, 5 840 individuals (AD and HE 264, AD no HE 246, HE no AD 1 085, no AD or HE 4 245) responded that they did not wish to disclose their annual household income. **‡‡** Individuals responding "unknown" were excluded as missing answers.  §§ Of these missing answers, 2 720 individuals (AD and HE 134, AD no HE 88, HE no AD 548, no AD or HE 1 950) responded that they did not wish to disclose their annual personal income. | | | |

| Missing data for Supplementary table 4a and 4b | | |
| --- | --- | --- |
|  | **Answers** | **Missing answers†,  total (AD and HE, AD no HE, HE no AD)** |
| **Current face and/or ear eczema** | 8 922 | 1 821 (180, 294, 1 347)‡ |
| **Current scalp eczema** | 8 727 | 2 016 (243, 330, 1 443)‡ |
| **Current neck and/or upper chest eczema** | 8 691 | 2 052 (223, 345, 1 484)‡ |
| **Current armpit eczema** | 8 533 | 2 210 (254, 354, 1 602)‡ |
| **Current arm eczema** | 9 068 | 1 675 (146, 245, 1 284)‡ |
| **Current hand eczema** | 10 616 | 127 (18, 0, 109)§ |
| **Current abdomen eczema** | 8 534 | 2 209 (274, 369, 1 566)‡ |
| **Current back eczema** | 8 507 | 2 236 (278, 378, 1 580)‡ |
| **Current inguinal eczema** | 8 442 | 2 301 (275, 385, 1 641)‡ |
| **Current buttocks eczema** | 8 439 | 2 304 (284, 385, 1 635)‡ |
| **Current genital eczema** | 8 353 | 2 390 (302, 392, 1 697)‡ |
| **Current leg eczema** | 8 863 | 1 880 (191, 297, 1 392)‡ |
| **Current foot eczema** | 8 578 | 2 165 (265, 391, 1 509)‡ |
| † Of the 10 743 individuals responding to the question on lifetime prevalence of physician diagnosed AD, lifetime prevalence of hand eczema, and lifetime prevalence of eczema in general, with either AD and HE (n=1 914), AD no HE (n=7 158), or HE no AD (n=1 671).  ‡ The question regarding localization of eczema was branched in a way that only individuals answering “Yes” to lifetime eczema was presented with the question. Individuals answering “No” to lifetime prevalence of eczema was assigned “No” to current eczema in a given localization. In a similar way, individuals answering “Never”, “Not currently but within past 12 months” or “For more than 12 months ago” regarding eczema in each localization was assigned “No” to current eczema in that localization. § Current hand eczema was defined as individuals responding “Yes” to point prevalence of hand eczema. No current hand eczema was defined as individuals responding “No” to lifetime, one year or point prevalence of hand eczema. | | |
